# DNN-MVL: DNN-Multi-View-Learning-Based Recover Block Missing Data in a Dam Safety Monitoring System

**DOI:** 10.3390/s19132895

**Published:** 2019-06-30

**Authors:** Yingchi Mao, Jianhua Zhang, Hai Qi, Longbao Wang

**Affiliations:** College of Computer and Information, Hohai University, Nanjing 211100, China

**Keywords:** missing data completion, multi-view learning, information fusion, DNN, dam safety monitoring, sensor networks

## Abstract

Many sensor nodes have been widely deployed in the physical world to gather various environmental information, such as water quality, earthquake, and huge dam safety. Due to the limitation in the batter power, memory, and computational capacity, missing data can occur at arbitrary sensor nodes and time slots. In extreme situations, some sensors may lose readings at consecutive time slots. The successive missing data takes the side effects on the accuracy of real-time monitoring as well as the performance on the data analysis in the wireless sensor networks. Unfortunately, existing solutions to the missing data filling cannot well uncover the complex non-linear spatial and temporal relations. To address these problems, a DNN (Deep Neural Network) multi-view learning method (DNN-MVL) is proposed to fill the successive missing readings. DNN-MVL mainly considers five views: global spatial view, global temporal view, local spatial view, local temporal view, and semantic view. These five views are modeled with inverse distance of weight interpolation, bidirectional simple exponential smoothing, user-based collaborative filtering, mass diffusion-based collaborative filtering with the bipartite graph, and structural embedding, respectively. The results of the five views are aggregated to a final value in a multi-view learning algorithm with DNN model to obtain the final filling readings. Experiments on large-scale real dam deformation data demonstrate that DNN-MVL has a mean absolute error about 6.5%, and mean relative error 21.4%, and mean square error 8.17% for dam deformation data, outperforming all of the baseline methods.

## 1. Introduction

Many wireless sensor networks (WSNs) have been widely deployed in the physical world to sense and collect various environmental information or events, such as water quality [1], air quality [2], forest fire [3], and dam safety [4]. These sensors generate massive geo-tagged time series data, helping humans to make further analysis and decision [5]. However, affected by hardware and severe wireless conditions, such as strong fade in WSNs, raw sensory data can have loss and corruption. Due to the limitation in the battery power, memory, and computational capacity, the readings generated from sensors are unreliable or inaccurate [6,7,8,9]. In addition, some sensors may malfunction and result in faulty and missing data in the unattended situations. Massive missing readings in the WSNs will not only affect real-time monitoring especially for emergency conditions, but also compromise the performance of further data analysis and decision. It is extremely important to obtain the full and accurate monitoring data from raw readings before making any further analysis and decision. Considering the internal and external factors, events occurred in the physical world cannot be accurately detected using inaccurate and incomplete sensory data [10]. Therefore, it is necessary to complete the missing reading from a large-scale wireless sensor network with geo-sensory time series data.

Several solutions have been proposed to handle block missing data, such as K-nearest neighbors-based local interpolation [11], Kriging interpolation [12], and multi-channel singular spectrum analysis (MSSA) based on PCA (Principal Component Analysis) [13]. Unfortunately, those methods cannot well establish the spatial-temporal correlations, which results in the fair-quality interpolation. Moreover, matrix completion (MC) methods have been applied to fill the missing reading from partially observed geo-sensory data [14,15]. Different the interpolation methods, matrix completion often seeks to find the lowest rank matrix or well matches the known entries of a matrix with missing data. Matrix completion techniques can be utilized for filling the random missing readings. However, when there exist block missing readings along the column or the row due to sensor failures, it is very difficult to find stable inputs for the matrix completion model. Some global initialization methods have been proposed to avoid the unstable inputs for the matrix completion. For example, non-negative matrix factorization based on the multi-view [16,17], the user-based collaborative filtering [18], and matrix factorization and a salient version called empirical orthogonal functions model [19,20,21,22] can be applied to fill missing values. Although those methods consider the spatial-temporal views, they just adopt the linear fusion model with multiple views results, which cannot generate more accurate estimate for filling missing data.

**Figure 1:**
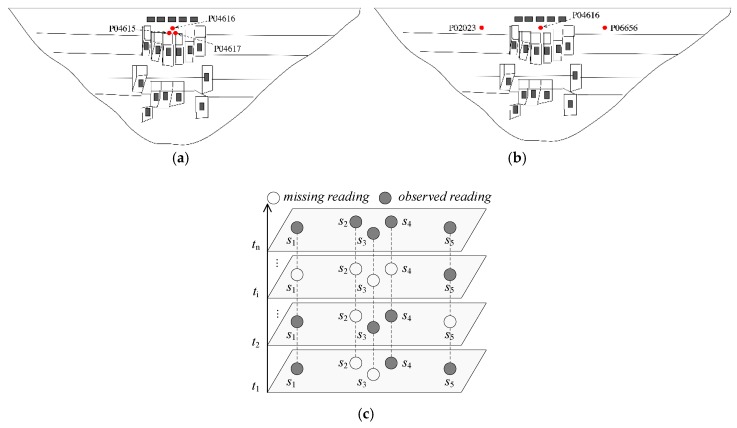
Illustration of sensor locations of dam deformation data. (**a**) Densely-deployed sensors; (**b**) loosely-deployed sensors; (**c**) missing readings situation.

Missing data can occur at arbitrary sensor nodes and time slots. In extreme situations, some sensors may lose readings at consecutive time slots. In this paper, the dam deformation observation dataset is from the real Dam Safety Monitoring Systems, recording the observation values from 2017/01/01 to 2017/12/31. There are five sensor nodes for monitoring the dam deformation P02023, P04615, P04616, P04617, and P06656, represented as $S_{1}$, $S_{2}$, $S_{3}$, $S_{4}$, and $S_{5}$, respectively shown in Figure 1. From Figure 1a,b, three sensors P04616, P04615, P04617 are closely deployed in the spatial space, while the spatial distribution between two sensors P02023, and P06656 is far away. As illustrated in Figure 1c, four sensor nodes lost their readings in the time slot $t_{i}$. Meanwhile, sensor node $S_{2}$ cannot record the observed readings from the time slot $t_{1}$ and $t_{i}$, which is called as block missing.

**Figure 2:**
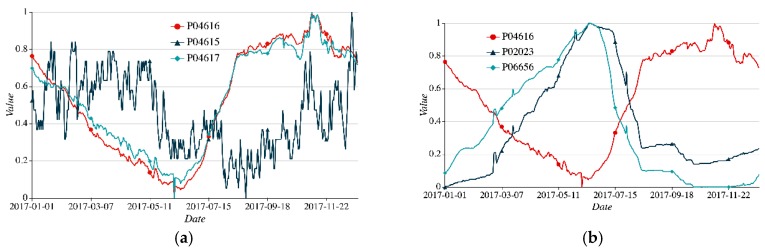
Illustrations of sensor readings of dam deformation data. (**a**) Densely-deployed sensors; (**b**) loosely-deployed sensors.

On the other hand, affected by multiple complex factors, sensor readings vary with the locations and time significantly and non-linearly. The observed data of sensors with a shorter distance may not always be more similar than those with a farther distance. For example, Figure 2 shows the observed data of five sensors from 1 January 2017 to 31 December 2017. From Figure 2a,b, the distance between two sensors P02023 and P06656 is much farther than the distance between two sensors P04615 and P04617. However, the readings of P02023 and P06656 are more similar than those of P04615 and P04617. The reason is that two sensors P02023 and P06656 are in two different regions with similar mechanical structure. This case violates the First Law of Geography: “near things are more related than distant things.” Furthermore, sensor readings sometimes exhibit a sudden change, as illustrated in Figure 2. The readings of sensor P02023 from 10 August 2017 to 14 August 2017. Such a sudden change may have a side effect on the real-time monitoring and data analysis. Existing interpolation-based and smoothing methods cannot obtain good results.

Filling block missing reading from a large-scale sensor network with geo-sensory time series data is a challenging task. To solve the above problem, a unified multi-view-learning model in a deep learning model (DNN-MVL) is proposed to fill the block missing values that jointly consider the spatial relations between different time series from the different sensor nodes, and the temporal relations between readings at different time slots in the same series from one node, and semantic correlations through graph embedding. We conducted extensive experiments on real dam deformation monitoring dataset and Beijing air quality dataset. The results demonstrate that the proposed method DNN-MVL can achieve high filling accuracy in the presence of block missing data.

The rest of this paper is organized as follows. We introduce the related work in Section 2. The preliminary and problem statement are presented in Section 3. The main framework of the proposed DNN-MVL is introduced in Section 4. In Section 5, we present our proposed DNN-MVL approach for filling the block missing data. Finally, we evaluate the performance of the proposed DNN-MVL with Beijing air quality data and large-scale real dam deformation data in Section 6, and conclude the work in Section 7.

## 2. Background and Related Work

Data missing is unavoidable problem during the data transmission and collection in the wireless sensor networks. To solve the data missing problem, existing solutions can be classified into two types: (1) the missing data is deleted from the time series. (2) The missing data is filled with an estimation with the interpolation model based on the historical data. It is a simple way to delete the missing readings from the time series. However, it may result in the poor data quality when deleting successive data missing. The missing reading filling is a better approach with interpolation or matrix completion.

A lot of work has been focused on the interpolation for the missing data. K-nearest-neighbor (KNN) [23] is a simple local interpolation method that utilizes the values of the nearest K neighbors to estimate its missing value. KNN is only applied for data interpolation with very few missing values. Unfortunately, it performs poorly for the blocking missing. Meanwhile, the sensory data from WSNs are usually the time series. The temporal correlation model can be used to fill the missing data. Auto-regressive integrated moving average model (ARIMA) is a well-known time series prediction method, which combines moving average and autoregressive components [24]. ARIMA is suitable to predict a missing value just solely considering a sensor’s readings [25]. In addition, sensor nodes are deployed in the different locations. Different geo-tagged time series data exhibit the spatial correlation. Inverse distance weighting (IDW), linear regression, and Kriging [26,27] are widely used spatial models. For example, IDW is applied to estimate the rainfall distribution [28], and Kriging is used to fill the missing temperature [29]. Those models can be used to interpolate a missing value with available readings in the spatial correlation. While those studies still fail to capture the complex non-linear spatial-temporal correlations.

Besides the above methods, matrix completion (MC) methods have emerged and been applied to fill the missing reading from partially observed geo-sensory data [30,31]. Different from the interpolation methods, matrix completion often seeks to find the lowest rank matrix or, if the rank of the completed matrix is known, which well matches the known entries of a matrix with missing data. Matrix completion techniques can be utilized for filling the random missing readings due to the unreliable wireless transmissions, if exploiting the spatial-temporal correlations. However, when there exist block missing readings along the column or the row due to fading or sensor failures, it is very difficult to find stable inputs for the matrix completion model.

Recently, there are many data-driven methods from multiple views for filling missing values were proposed. To model the spatial-temporal correlation, a data estimation for mobile sensors method was presented to fill the missing data through establishing the time-dependent spatial-temporal correlation in the mobile wireless sensor networks [32,33]. When there is not enough spatial and temporal information, a spatial and temporal K-nearest neighbors (ST-KNN) imputation method adopted the weighted average to fill the missing values [34]. In the recommendation system, user-based and item-based collaborative filtering methods were introduced to fill missing values considering the users similarity and temporal similarity [35]. However, when meeting the sparse matrix problem, it is difficult to generate the accurate the filling values due to the inaccurate results of similarity. Considering spatial, temporal, global and local views, combining the statistic models and data-driven models in a spatial–temporal multi-view learning (ST-MVL) framework was established to generate a more accurate estimate [20]. These studies on the multiple view learning methods are mainly based on the linear fusion from different views to estimate the missing values. They fail to model the complex non-linear relations of the space and time.

In summary, the difference of our proposed method DNN-MVL compared with other work is that we consider the spatial correlation, temporal sequence correlation and functional correlation in a joint deep learning model.

## 3. Problem Statement

**Figure 3:**
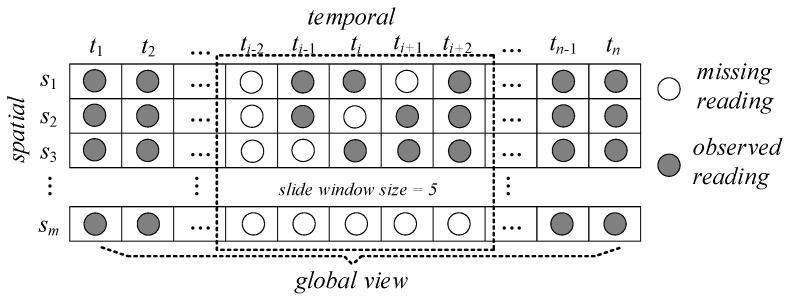
Data matrix of sensor readings.

In this section, we first fix some notations and present the problem formulation. In the WSNs, the data collected from the monitoring of the dynamic environment can generally be represented by a matrix, defined as $M_{S\times T}$. The matrix $M_{S\times T}$ records data from S sensors over T time slots, in which $S=\{s_{1},\ldots ,s_{i},\ldots ,s_{n}\}$ represents the n sensors’ readings and $T=\{t_{1},\ldots ,t_{j},\ldots ,t_{m}\}$ represents the m consecutive time slots. As shown in Figure 3, in the matrix $M_{S\times T}$, a row stands for a sensor and a column denotes a time slot. An entry $m_{i,j}$ represents the readings of the sensor $s_{i}$ at the time slot $t_{j}$. Due to hardware failure or severe transmission condition in the WSNs, the measured and collected data are often unreliable. As a result, both the matrix rows and columns in the sensory matrix $M_{S\times T}$ may have some successive missing data, as shown in Figure 3. In this paper, we call it a block missing problem. The filling missing data problem aims to fill the readings at time interval given the data until the time interval.

Since the observed data in WSNs normally have strong correlation between neighboring locations and timestamp, we can incorporate temporal views, spatial views, and functional (semantic) views to fill the missing readings of sensors. Therefore, in order to fill the block missing readings, we propose DNN-MVL method to collectively consider the global-local, spatial-temporal, and semantic views simultaneously to fill the missing readings. The results of the five views are aggregated to a final value in a multi-view learning algorithm with DNN model.

## 4. Main Idea

To capture the complex and non-linear spatial-temporal correlations when filling the block missing values, DNN-based multiple view learning framework was proposed. The temporal view establishes the sequential relations model in the time series and estimates the missing values of a sensor based on the readings at the neighbor time slots of the same sensor node. The readings of sensors are often smooth in a small spatial area. The recorded data at nearby locations have similar values at a given time. The spatial view can estimate a missing value based on the spatial similarity between a sensor’s current readings and those of sensors’ spatial neighbors. Furthermore, different time length of the observed data for filling missing readings can be considered as global view and local view. For example, if an entry $m_{i,j}$ is lost, we can use the adjacent readings of sensor $s_{i}$ from $t_{j-2}$ to $t_{j+2}$ to fill the missing readings, which is called as a temporal local view. Likewise, we also estimate the reading of $m_{i,j}$ based on the its spatial neighbors $s_{i-1}$ and $s_{i+1}$, which is a spatial local view. Obviously, local view can capture the instantaneous changes. We can consider the readings over a long time period from $t_{1}$ to $t_{m}$ to recover the missing value, which is regarded as a global view. Global views can represent the long-term patterns. In general, the sensors with similar functionality may have similar patterns, e.g., at both ends of the arch dam may have similar structural mechanics. Similar regions may not necessarily be close in space. Therefore, the structural embedding method is applied to construct a graph of sensors’ locations representing functional similarity among the sensors in the different regions. In this paper, semantic similarity is equal to functional similarity.

**Figure 4:**
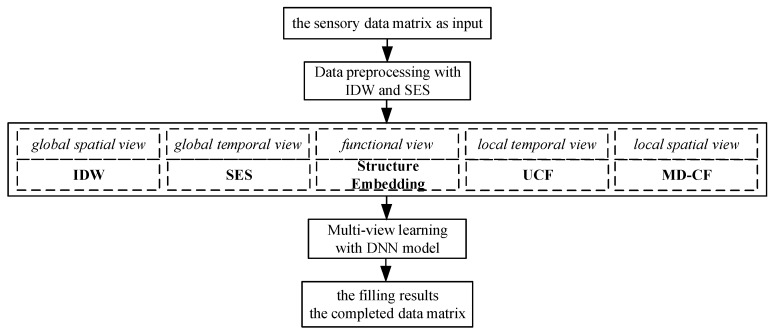
Framework of proposed DNN-multi-view learning method (MVL) method.

Our proposed DNN-MVL framework includes five views, as shown in Figure 4. Global spatial view with inverse distance weighting interpolation can compute an estimated value of a missing reading based on the values of the sensors’ spatial neighbors. Global temporal view with bidirectional simple exponential smoothing is used for analysis of time series data. Exponential smoothing is a prediction model derived from amount of historical data using the exponential window function to assign exponentially decreasing weights over time. From the local spatial view, if a sensor node is regarded as a user, user-based collaborative filtering can predict the missing reading based on the local similarity between a sensor’s readings and those of its neighbors. Local temporal view with mass diffusion-based collaborative filtering method can estimate the missing readings based on the local similarity between recent readings at the different time slots of the same sensor. Semantic view is constructed with a graph of sensors’ locations representing the functional similarity among different locations. With the dynamic time wrapping method, the functional similarity is measured by the similarity between the time sequences originated from two sensor nodes. To leverage the results of different views for the missing readings, a deep neural network model is adopted to generate the final results of five different views’ estimations with minimal errors.

## 5. DNN-Based Multiple View Learning Framework

In this section, we provide details of the proposed DNN-based multiple view learning framework (DNN-MVL) for filling the block missing values. Our proposed DNN-MVL framework includes five views: global temporal view with IDW, local temporal view with simple exponential smoothing (SES), local spatial view with user-based collaborative filtering (UCF), local temporal view with mass diffusion (MD)-CF, and semantic view with structural embedding. The results of the five views are aggregated to obtain the final filling readings with DNN model.

### 5.1. Global Spatial View: IDW

To capture the long-term patterns in spatial relations, the inverse distance weighting interpolation (IDW)—a statistical model, is applied to interpolate a missing value based on its spatial neighbor sensors. IDW is a deterministic method for multivariate interpolation with a known scattered set of points. The closer the sensor with the available readings is to the target node, the greater the weight assigned to it. The assigned values to the missing values are calculated with a weighted average of the available readings of geospatially adjacent sensors. IDW estimates the prediction value *m_gs_* can be calculated as follows:

(1) $$m_{gs}=\frac{\sum_{i=1}^{n}m_{i,a}\times d_{i}{}^{-\alpha }}{\sum_{i=1}^{n}d_{i}{}^{-\alpha }},$$

where $m_{i,a}$ is the value of sensor $s_{i}$ at the time slot $t_{a}$, and n is the number of sensors participating in filling missing data. $d_{i}$ is the spatial distance between a candidate sensor and the target sensor. α is a positive parameter that affects the decay rate of a sensor’s weight by the geospatial distance. The value of α varies from 0 to 1. The closer sensor’s readings can perform better filling results for a missing reading. $d_{i}{}^{-\alpha }$ assigns a bigger weight to closer sensor’s readings. Otherwise, the farther sensor’s readings are assigned with a smaller weight. $m_{gs}$ is filling results with global spatial view.

To further illustrate the spatial correlation in the different sensors’ readings, the similarity ratio $sim_{i,j}$ between arbitrary two sensors’ readings at the same time slot can be calculated as follows:

(2) $$sim_{i,j}=1-\frac{\left|m_{i,a}-m_{j,a}\right|}{dist_{i,j}},$$

where $m_{i,a}$ and $m_{j,a}$ are the readings of sensor $s_{i}$ and sj at the time slot $t_{a}$ respectively, $dist_{i,j}$ is the geospatial distance between two sensors $s_{i}$ and sj.

**Figure 5:**
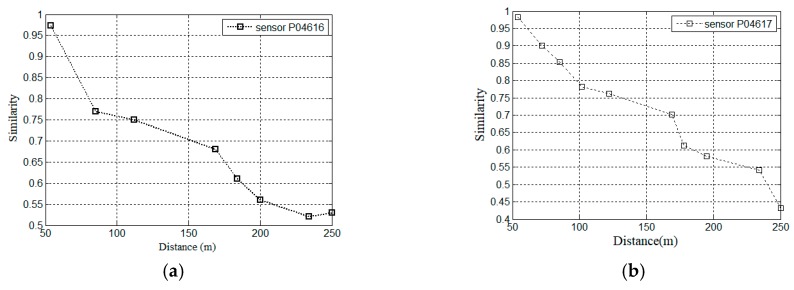
Spatial similarity in different sensors’ readings. (**a**) Sensor P04616; (**b**) sensor P04617.

Figure 5a,b show the similarity ratio between two sensors P04616, P04617 and other deformation sensors in the dam safety monitoring dataset, respectively. The similarity ratio decreases as the distance between two sensors increases in most cases, which actually follows the First Law of Geography. i.e., “everything is related to everything else, but near things are more related than distant things,” which is an empirical spatial correlation in geo-sensory data. Two other sensors, P04615 and P04617, are near target sensor P04616, with geospatial distance 54 m and 64 m respectively. If it set the decay rate α=1, the assigned weights to two sensors are 1/54 and 1/64. We can calculate an estimation $m_{gs}=54.2$ with the weighted average values by Equation (1). We can estimate the missing values with Equation (1). To further illustrate the spatial correlation in the different sensors’ readings, Equation (2) is adopted to measure the similarity of two sensors in the global spatial correlation. Two equations can compute the spatial similarity from the different aspects. That motivates us to apply IDW interpolation to model the global spatial view. However, not all the sensor nodes are farther away from the target sensor, their similarities are smaller. For example, Figure 2b illustrates that the sensor P02023 is far away from sensor P06656. They have similar data pattern. In this paper, other views should be considered to obtain more accurate estimation.

### 5.2. Global Temporal View: SES

To consider the global temporal view, SES is utilized to estimate the missing value based on the historical data of same sensor at other time slots. SES is a prediction model in the time series domain using the exponential window function. SES computes the prediction value $m_{gt}$ as follows:

(3) $$m_{gt}=\sum_{j=1}^{s}\beta {\left(1-\beta \right)}^{j-1}m_{b,s-j+1},$$

where $t_{s}$ is the time slot of the missing reading, $t_{j}$ is a time interval between a candidate reading $m_{b,s-j+1}$ of sensor $s_{b}$ and a target reading $m_{b,s}$; β is a smoothing parameter with a range of (0, 1). In general, if the time interval is smaller to the target one, $\beta {\left(1-\beta \right)}^{j-1}$ will be set to a bigger weight. A smaller β means a slower decay of weight over the time interval. However, the traditional SES only uses historical readings of the target time slot as input to model average exponential moving. In our method, it considers both the historical readings and successive readings of a target time slot as inputs to compute the average weighted smoothing.

Given a target time slot t, the observed reading of sensor $s_{b}$ is $m_{b,t}$. SES assigns a weight $\beta \times {\left(1-\beta \right)}^{\left|t_{x}-t\right|}$ to each reading of the same sensor $s_{b}$ at the candidate time slot $t_{x}$, where $\left|t_{x}-t\right|$ is the time interval between the candidate time slot and target slot. Thus, the prediction value $m_{gt}$ using SES can be calculated as follows:

(4) $$m_{gt}=\frac{\sum_{x=1}^{m}\beta \times {\left(1-\beta \right)}^{\left|t_{x}-t\right|}\times m_{b,t_{x}}}{\sum_{x=1}^{m}\beta \times {\left(1-\beta \right)}^{\left|t_{x}-t\right|}}.$$

**Figure 6:**
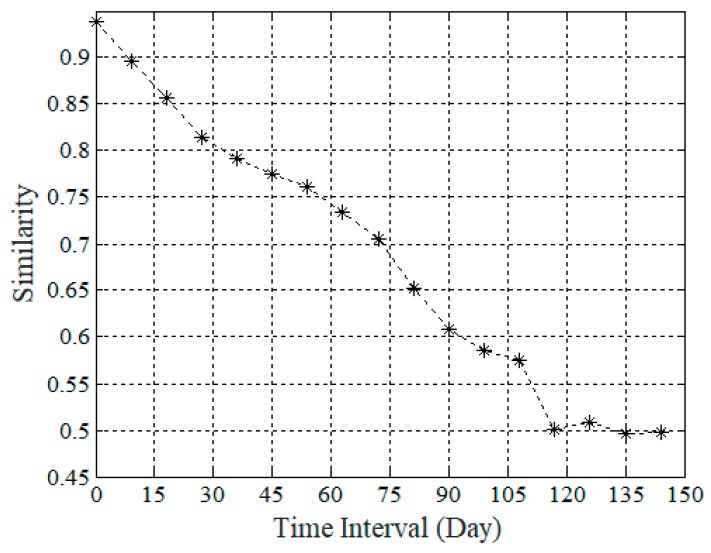
Temporal similarity in a sensor’s readings.

The SES model is inspired by the observation from time series data. Figure 6 shows the similarity ratio between arbitrary two readings at two different time slots of the same sensor in the dam deformation dataset. Each point in Figure 6 denotes the similarity ratio between the arbitrary reading and the target reading at the given time interval in the sensor P04616. As shown in Figure 6, the curve of similarity ratio decreases as the time interval increases. It is an empirical temporal correlation in time series. The readings of recent time slots are more relevant than those of distant time slots.

We use the example of dam deformation data to clearly demonstrate the performance of SES model. Suppose there is a missing reading at time slot $t_{x}$, and the observed readings at the four adjacent time slots $\left(t_{x-2},t_{x-1},t_{x+1},t_{x+2}\right)$ are (85, 75, 75, 85) respectively. If the smoothing parameter β is set to 0.5, the weights for four time slots are (0.25, 0.5, 0.5, 0.25). The prediction value $m_{gt}$ is 78.3 by Equation (4).

### 5.3. Local Spatial View: UCF

The fluctuation of dam deformation data is often smooth in a small region at a given time interval. The observed readings at nearby locations are similar. The local spatial correlation between a sensor node $S_{c}$ and its neighbors in a time slot $t_{x}$ can be measured as follows:

(5) $$LS_{gap}(s_{c},t_{x})=m_{c,j}-N_{\left(c\right)}M^{\left(x\right)}/\sum N_{\left(c\right)},$$

where $N_{\left(c\right)}$ is the c-th row of topology matrix N, $M^{\left(x\right)}$ is the x-th column of sensory matrix M. The topology matrix N is defined as follows:

(6) $$N={\left(N_{\left(i,j\right)}\right)}_{n\times n}=\{\begin{array}{cc} 1 & if\;i\;and\;j\;are\;1-hop\;neighbors\\ 0 & otherwise \end{array}.$$

With the locations of all deployed sensor nodes, it can be easily to obtain the topology matrix. In Equation (5), $N_{\left(c\right)}M^{\left(x\right)}$ is the total observed value of the neighbors of sensor $s_{c}$, and $N_{\left(c\right)}$ is the number of $s_{c}$’s one-hop neighbors. $N_{\left(c\right)}M^{\left(x\right)}/\sum N_{\left(c\right)}$ means the average data value of one-hop neighbors of sensor $s_{c}$ at the time slot $t_{x}$. Equation (5) can compute the difference between the reading of sensor $s_{c}$ and the average readings of its one-hop neighbors at a given time slot. Each element $N_{\left(i,j\right)}$ in the matrix N represents whether the sensors $s_{i}$ and $s_{j}$ are one-hop neighbors. It is obvious that topology matrix N has binary values to represent the relationship between two sensor nodes.

To further capture the spatial correlation between sensor nodes, UCF is motivated to model the spatial local correlation. The main idea of UCF is that similar users usually make similar scores for similar items. In the dam deformation monitoring, each sensor is regarded as one user, and one deformation reading of one sensor at a time slot is regarded as one item. The window size ω is adopted to normalize the different scale of different sensor nodes. The local readings matrix for sensor node $s_{u}$ and $s_{v}$ from the time slot t−(ω−1)/2 to t+(ω+1)/2 are $\left[m_{u,t-(\omega -1)/2},\ldots ,m_{u,t+(\omega +1)/2}\right]$ and $\left[m_{v,t-(\omega -1)/2},\ldots ,m_{v,t+(\omega +1)/2}\right]$, respectively. The similarity measurement between two sensors $s_{u}$ and $s_{v}$ can be computed based on the cosine vector as follows:

(7) $$sim(s_{u},s_{v})=\frac{\sum_{i\in I_{uv}}\left(m_{u,i}-\bar{M_{\left(u\right)}})(m_{v,i}-\bar{M_{\left(v\right)}}\right)}{\sqrt{\sum_{i\in I_{u}}{\left(m_{u,i}-\bar{M_{\left(u\right)}}\right)}^{2}\times \sum_{i\in I_{v}}{\left(m_{v,i}-\bar{M_{\left(v\right)}}\right)}^{2}}},$$

where $m_{u,t}$ and $m_{v,t}$ are the observed readings of sensor $s_{u}$ and $s_{v}$ at the time slot t, $\bar{M_{\left(u\right)}}$ and $\bar{M_{\left(v\right)}}$ are the average values of two sensors respectively; $I_{u}$ and $I_{v}$ are two time vectors of two sensors that there is no missing readings; $I_{uv}$ is the time vector that two sensors $s_{u}$ and $s_{v}$ both have readings, that is $I_{uv}=I_{u}\cap I_{v}$.

The sensors’ similarities are calculated and sorted in descending order by Equation (7). The most similar k target sensors are selected to construct the set of the nearest neighbors V, where $sim(s_{u},s_{v_{1\cdots k}})>sim(s_{u},s_{v_{k+1\cdots m}})$ and $V=\{v_{1},\ldots ,v_{k}\}$. Then, we can calculate an estimation $m_{ls}$ from local spatial view with the weighted average similarity as a weight as follows:

(8) $$\overset{}{m_{ls}}=\frac{\sum_{u=1}^{k}m_{u,t}\times sim(s_{u},s_{v})}{\sum_{u=1}^{k}sim(s_{u},s_{v})}.$$

IDW does not hold the sudden change in global spatial correlation, e.g., the case shown in Figure 2b, while UCF can have good performance on the local spatial correlation by capturing the time-dependent spatial correlation in a small area between sensors’ readings.

### 5.4. Local Temporal View: MD-CF

In general, dam deformation data usually change slowly over time. To study the short-term and local temporal correlation for one sensor, we calculate the gap between each pair of adjacent readings for the same sensor in two consecutive time stamps $t_{j}$ and $t_{j-1}$ as follows:

(9) $$T_{gap}(i,j)=|m_{i,j-1}-m_{i,j}|$$

where $m_{i,j}$ represents the observed data of sensor $s_{i}$ at the time slot $t_{j}$. If the observed reading is not changed from the time slot $t_{j-1}$ to $t_{j}$, it has $T_{gap}(i,j)=0$. The smaller $T_{gap}(i,j)$, the more stable the observed readings for sensor $s_{i}$ at the time slot $t_{j}$.

However, sensor readings sometimes exhibit a sudden change, as illustrated in Figure 2. The readings of sensor P02023 from 10 August 2017 to 14 August 2017. In addition, the block missing of data results in the data sparsity. It is hard to calculate the data similarity from the adjacent readings for the same sensor. To fill the missing readings from the local temporal view of the same sensor, MD is introduced to item-based collaborative filtering for estimating the missing readings, where a time slot denotes an item. MD refers to the movement of matter from place to place, resulting in a net change in the mass’s location [36]. MD is an extremely common phenomenon in everyday life.

**Figure 7:**
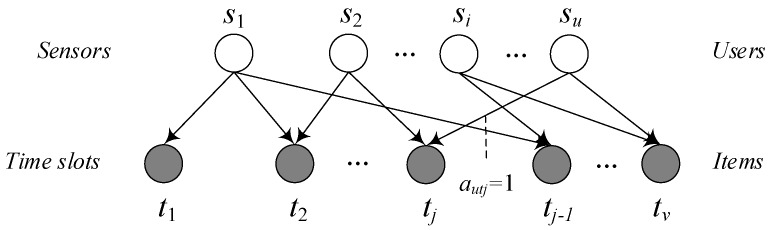
The bipartite graph of sensors and time slots in mass diffusion (MD)-collaborative filtering (CF).

With bipartite graph, mass diffusion method can be applied to fill the missing data. In the MD-CF method, bipartite graph represents the relationship between users and items. In the bipartite graph, sensor node denotes the user and a time slot denotes an item, as shown in Figure 7. The set of sensor nodes is denoted as $S=\{s_{1},\ldots ,s_{i},\ldots s_{u}\}$ and the set of time slots is $T=\{t_{1},\ldots ,t_{j},\ldots t_{v}\}$. If the reading of sensor node $s_{u}$ at the time slot $t_{j}$ is not missing, there has an edge between the node $s_{u}$ and $t_{j}$. That is $a_{ut_{j}}=1$. Otherwise, $a_{ut_{j}}=0$. Thus, the bipartite graph can represent whether the sensor nodes have the missing readings at the different time slots. Through the iteration of mass diffusion, it can seek the degree of association between two nodes in the bipartite graph [37], and then it can calculate the similarity of the readings at the different time slots. The specific steps are as follows:

(1) Initialization phase. Assume that $m_{u,t_{i}}$ denotes the reading of node $s_{u}$ at the time slot $t_{i}$, and $\bar{M_{\left(u\right)}}$ is the average value of readings from the node $s_{u}$. The initial energy $e_{0}$ at the time slot $t_{i}$ can be calculated as follows:

(10) $$e_{0}=\frac{1}{1+\sum_{u=1}^{m}|m_{u,t_{i}}-\bar{M_{\left(u\right)}}|\times a_{ut_{i}}}$$

(2) Energy diffusion from time nodes to sensor nodes. The energy of sensor node $s_{u}$ are equally diffused to other sensor nodes which have readings at the time slot $t_{i}$. The energy of sensor node $s_{u}$ at the time slot $t_{i}$ is denoted as $e_{t_{i}u}$, which can be calculated as follows:

(11) $$e_{t_{i}u}=a_{u,t_{i}}/k(t_{i})$$

where $k(t_{i})$ is the degree of time node $t_{i}$ in the bipartite graph, and $k(t_{i})$ refers to the number of sensor nodes which have the readings at the time slot $t_{i}$. If the sensor node $s_{u}$ has readings at the time slot $t_{i}$, it has $a_{ut_{i}}=1$. Otherwise, $a_{ut_{i}}=0$.

(3) Energy diffusion from sensor nodes to time nodes. The energy of sensor node $s_{u}$ are equally diffused to time nodes where sensor nodes have readings at the time slot $t_{j}$ based on the degree of sensor node $s_{u}$, in which $t_{j}>t_{i}$. The final energy of time node $t_{j}$ is the sum of diffused energy from all sensor nodes connected with the time node $t_{j}$. After twice energy diffusions, the similarity of two different time slots $t_{i}$ and $t_{j}$ for the same node $s_{u}$ can be computed with the proportion of the obtained energy from the time slot $t_{i}$ to $t_{j}$. The similarity of two different time slots $sim(t_{j},t_{i})$ can be computed as follows:

(12) $$sim(t_{j},t_{i})=\sum_{u\in S}\frac{a_{ut_{j}}\times e_{i,u}}{k(u)}=\frac{e_{0}}{k(t_{i})}\sum_{u\in S}\frac{a_{ut_{j}}\times a_{ut_{i}}}{k(u)},$$

where k(u) is the degree of sensor node $s_{u}$ in the bipartite graph, the value of $e_{t_{i}u}$ can be calculated with Equation (11).

(4) If the missing reading at the time slot $t_{i}$, the k adjacent time slots of $t_{i}$ are selected. Then, the similarities of all pairs of two different time slots in are calculated by Equation (12) and sorted in descending order, denoted as $T=\{t_{1},\ldots ,t_{j},\ldots t_{k}\},t_{i}\notin T$. That is $sim(t_{i},t_{1})>\ldots sim(t_{i},t_{j})>\ldots >sim(t_{i},t_{k})$.

(5) Adopting the CF algorithm, the weights are assigned based on the similarities. It can obtain the filling result from the local temporal view, as follows:

(13) $$m_{lt}=\sum_{t\in T}sim(t_{i},t)\times m_{u,t}/\left|\sum_{t\in T}sim(t_{i},t)\right|.$$

MD-CF method is applied to establish the time-dependent local temporal correlation learned from recent data as well as avoid the low accuracy due to the block missing.

### 5.5. Semantic View: Structural Embedding

Intuitively, sensor nodes sharing similar functionality may have similar data distribution. However, sensors with similar functionality may not necessarily be close in space. For example, although the spatial distance between two sensors P02023 and P06656 is not close, shown in Figure 1 and Figure 2, they have a similar data distribution. This is because their mechanical models exhibit symmetric structure. We establish a graph of sensors representing functional (semantic) similarity among sensors [38]. The semantic graph is defined as G=(V,E,D), where the set of nodes are sensors V=S, E is the edge set E∈V⊗V, and D is the set of functional similarity on all of the edges. Dynamic time warping (DTW) method [39] is applied to measure the functional similarity $\varphi_{i,j}$ between sensor $s_{i}$ and sensor $s_{j}$ as follows:

(14) $$\varphi_{i,j}=exp(-\gamma \times DTW(s_{i},s_{j})),$$

where γ is the decay parameter and $DTW(s_{i},s_{j})$ is the dynamic time warping distance between the data distribution of two sensors $s_{i}$ and sensor $s_{j}$. In the dam safety monitoring systems, we use the average seasonal deformation time series as the dam deformation patterns. The average values can be computed based on the training data in the experiment.

To fill the missing readings from the sematic view, the graph embedding method -LINE [40] is applying to extract the feature vector $M_{i}$ of the data pattern for sensor $s_{i}$. In order to train the feature vector $M_{i}$, the feature vector is input to a fully connected layer. Thus, we can get the filling results $m_{se}$ with training model, which is defined as:

(15) $$m_{se}=f(W_{fe}\cdot M_{i}+b_{fe})$$

where two parameters $W_{fe}$ and $b_{fe}$ are both learning parameters.

### 5.6. Multi-View Learning with DNN

Each view has its own feature. It does not work well if purely using global, local, or semantic views. It may obtain the better filling results if adopting multiple views fusion. The proposed DNN-MVL method integrates the estimations of the above five views to generate the final result through a multi-view learning algorithm. The linear fusion is one of the simplest solutions, however, it cannot deal with the non-linear relation among the different estimations from five views. DNN-MVL applies the DNN-based multi-view learning to fuse different predictions.

Algorithm 1 presents the procedure of DNN-MVL. When a sensor network faces a block missing problem, local spatial and temporal views cannot separately work very well. First, global spatial and temporal views (IDW and SES) are applied to generate the initial values for those missing readings, respectively. Then, five different views are used to compute the estimations for each missing entry by using IDW, SES, UCF, MD-CF, and SE, respectively. Third, it aggregates the five estimations with DNN-based multi-view learning framework to calculate the final filling value. During the procedure of deep fusion, six-layer fully connected network is used as hiding layer, and the number of cell nodes in hiding layer is 32, 64, 256, 18, 64, and 32, respectively. The activation function in the hidden layer adopts ReLU function to reduce the gradient disappearance [41]. The batch normalization is adopted to accelerate the training and convergence before the ReLU function of each layer is activated [42]. Moreover, five-fold cross-validation is used to solve the problems of over-fitting and sparse training data. Five-fold cross validation can obtain the more reliable and stable model via avoiding the noise. In the output layer, a linear activation function is applied to compute the final filling result. In the DNN-MVL, the model is optimized for each sensor respectively by minimizing the least square error between estimations and the ground truth.

**Algorithm 1:** DNN_ multi-view learning method (MVL).**Input**: Original Data Matrix $M_{S\times T},\omega ,\alpha ,\beta ,t$; Structure Graph G=(V,E,D); **Output:** Final filling data matrix1. *O* ← Get-All-Missing-Values ($M_{S\times T}$);2. **If** there are successive missing readings ($M_{S\times T}$)3. $M_{S\times T}$ ← Initialization ($M_{S\times T},\alpha ,\beta$); 4. **For each** target sensor $s_{u}$ at the time slot t in *O*5.     $m_{ls}$ ← $UCF(M_{S\times T},t,\omega )$
6.     $m_{lt}$ ← $MD-CF(M_{S\times T},t,\omega )$
7.     $m_{gs}$ ← $IDW(M_{S\times T},t,\alpha )$
8.     $m_{gt}$ ← $SES(M_{S\times T},t,\beta )$
9.     $m_{se}$ ← $f(W_{fe}MM^{i}+b_{fe})$
10.     mm ← $DNN-MVL(m_{ls},m_{lt},m_{gs},m_{gt},m_{se})$
11.     Add mm into $M_{S\times T}$
12. **Return**
$M_{S\times T}$


## 6. Performance Evaluations

### 6.1. Datasets and Ground Truth

In this paper, the real dataset is used to evaluate the proposed DNN-MVL model. The dam safety monitor data from the highest arch dam in the world is from 2012/01/01 to 2017/08/14, which has 6159 timestamps respectively. In the experiments, 964 sensor nodes are selected from the dam safety monitor systems. Each of node generates a reading every four hours, as depicted in Figure 1. From the dataset, we fill the missing values whose statistics are shown in Table 1. There are two missing situations: block missing and general missing. The block missing is comprised of spatial block missing and temporal missing. The two-block missing may have some overlap. The spatial block missing is referred to as the reading values of all sensors simultaneously absent. The temporal block missing is the values of the same sensor with data absent in a certain temporal window size. As shown in Table 1, there are 4.6% of missing values in dam-deformation property when ω=11. General missing is the missing values except for the block missing. For example, about 15.2% of sensor readings in the Dam-Deformation dataset, including 8.4% general missing and 2.7% spatial block missing.

### 6.2. Data Preprocessing

In the experiment, the one-year data is partitioned into two parts. The data in March, June, September, and December are drop out as a testing set, and the rest are used for training. To train the proposed DNN-MVL framework, the local matrix from the training dataset is selected as the non-block missing data. Then, the well-trained model is applied to fill the block missing data. The values of the non-missing data in the testing set as the ground truth to evaluate the accuracy of our model. The dataset partition is shown in the Table 2.

### 6.3. Baselines

We compare our model with the following methods and tuned the parameter for all the methods.

Auto-regressive integrated moving average (ARIMA): ARIMA is a well-known model for forecasting time series which combines moving average and autoregressive components for modeling time series. ARIMA is fitted to predict a missing value based on the stationary time series. Seasonal ARIMA (SARIMA) considers the seasonal factors in time series.

Kriging: Kriging is a method of interpolation for which the interpolated values are modeled values are modeled by a Gaussian process governed by prior covariance. The method is widely used to interpolate a missing value with available readings in the domain of spatial analysis.

Data estimation for mobile sensors (DEMS): Gruenwald et al. proposed a method to predict the values based on the spatial-temporal correlations, considering the previous readings of a missing sensor and its neighbor’s readings at current time linearly.

Spatial-temporal K-nearest neighbor (ST-KNN): ST-KNN uses the weighted average readings of one sensor’s k nearest spatial and temporal neighbors to fill its missing values. For example, if k=6, the six nearest neighbors are selected based on the spatial and temporal model.

Collaborative filtering (CF): CF is a technique used by recommender systems. CF is a method of making automatic predictions (filtering) about the interests of a user by collecting preferences from many users (collaborating). In the experiment, every sensor is regarded as one user, and the readings of each sensor is regarded as the user’s preferences. CF is applied to generate a prediction and fill the missing reading based on the neighbor’s readings.

Spatial–temporal multi-view-learning (ST-MVL): Considering the temporal correlation between readings at different timestamps in the same series and the spatial correlation between different time series, a spatial-temporal multi-view-based learning method was proposed to collectively fill missing reading in a collection of geo-sensory time series.

We use mean absolute error (MAE), mean relative error (MRE), and mean square error (MSE) to evaluate our DNN-MVL framework, which are defined as follows:

(16) $$MAE=\frac{\sum_{i=1}^{\xi }\left|m_{i}-\hat{m_{i}}\right|}{\xi },\;MRE=\frac{\sum_{i=1}^{\xi }\left|m_{i}-\hat{m_{i}}\right|}{\sum_{i=1}^{\xi }\hat{m_{i}}},\;MSE=\frac{1}{\xi }\sum_{i=1}^{\xi }{\left(m_{i}-\hat{m_{i}}\right)}^{2},$$

where $m_{i}$ and $\hat{m_{i}}$ mean the prediction value and the ground truth for the time interval t+1, and where ξ is total number of samples.

### 6.4. Experimental Results Analysis

#### 6.4.1. Comparison with the Baseline Methods

Table 3 shows the performance of the proposed DNN-MVL as compared to all other methods, where DNN-MVL outperforms all other competing methods based on dam deformation dataset. From the experimental results, it can find that DNN-MVL can achieve the lowest MAE (13.54), MRE (0.19), and MSE (19.45) for filling the spatial block missing; and lowest MAE (9.75), MRE (0.156), and MSE (18.38) for filling the temporal block missing, among all the methods. More specially, we can see that ARIMA and SARIMA performed poorly, e.g., they had a MAE of 25.34 and 25.42 and an MSE of 29.11 and 30.24 when filling the spatial and temporal block missing values, respectively. The reason is that they just dependent on the historical spatial or temporal readings for prediction. DEMS, ST-KNN, and CF methods further consider the temporal–spatial correlation simultaneously, and therefore they can achieve better performance compared to ARIMA, Kriging, only relying on temporal or spatial relations, respectively. However, DEMS, ST-KNN, and CF methods do not model the global and local features. The proposed DNN-MVL achieved a lower MAE, MRE, and MSE compared to the best of the above three methods. The reason is that DNN-MVL combines the different views to predict the missing values through a non-linear multi-view learning way. Furthermore, DNN-MVL can obtain 6.5% (MAE), 21.4% (MRE), and 8.17% (MSE) relative improvement over the best performance among all baseline methods—ST-MVL in filling the spatial block missing values; and 7.60% (MAE), 22.64% (MRE), 10.10% (MSE) relative improvement for temporal block missing. Compared to ST-MVL, DNN-MVL adopts a semantic view to capture the functionality similarity, which are utilized to model the functional correlation among sensors. Therefore, DNN-MVL can significantly improve the effectiveness of filling the block missing readings.

**Figure 8:**
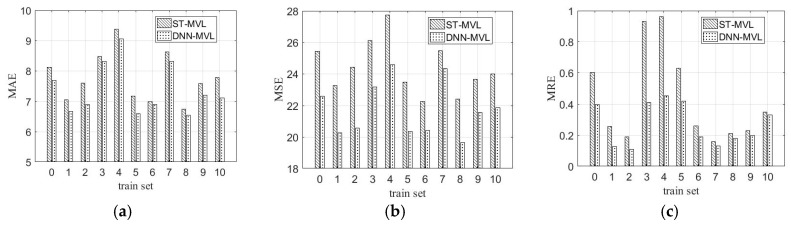
Comparisons in evaluations of training set. (**a**) Mean absolute error (MAE); (**b**) mean square error (MSE); (**c**) mean relative error (MRE).

To further evaluate the filling performance of both methods (DNN-MVL and ST-MVL) on the different datasets, the full dataset of dam deformation is divided into 11 training sets to compare the prediction values and ground truths. Figure 8a–c illustrates the MAE, MSE, and MRE results of DNN-MVL and ST-MVL in 11 different training sets. As shown in Figure 8a–c, DNN-MVL also outperforms the ST-MVL under all training sets.

#### 6.4.2. Results of Combination Methods

To further study the effect of different view components on the filling the missing readings, we also compare the performance of different views combination proposed in our method, as shown in Table 4.

Table 4 shows the results of different combinations from multiple views based on the dam deformation data. Our proposed DNN-MVL method can bring a significant improvement beyond the best single view SES and the best combination of two views (IDW + SES). Meanwhile, the combination methods from two views can have better performance than those with a single view. For example, the combinations of two views UCF and MD-CF can achieve higher accuracy than those with one single view UCF and MD-CF, respectively. In addition, UCF and MD-CF methods have better missing results than IDW and SES, respectively, which shows the effectiveness of the local dependency when filling the missing readings. ST-MVL can outperforms the global view and local view significantly, due to capturing the non-linear relations with the spatial-temporal view. Furthermore, DNN-MVL fuses the results from five views, including long-term patterns, knowledge-driven contexts, spatial correlation among different locations, and temporal correlation among different time slots. DNN-MVL can reduce the MAE 7.38% compared with ST-MVL. Our proposed method can exhibit the best performance on filling results.

**Figure 9:**
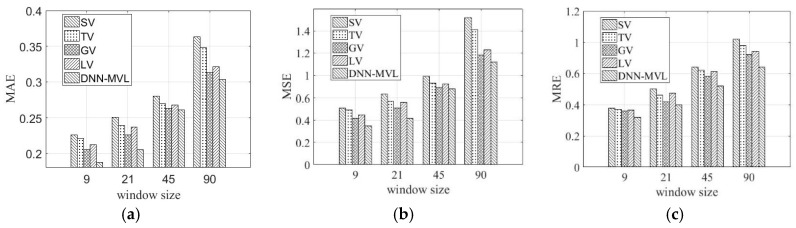
Performances of different views’ combinations. (**a**) MAE; (**b**) MSE; (**c**) MRE.

We study the filling performance on the window size of missing data sequence for five different views, as shown in Figure 9. Figure 9a–c shows the filling error of MAE, MSE, and MRE with respect to the window size ω, respectively. The window size is set to 9, 21, 45, and 90. A large window size may lose time dependency, but a small window size may not capture the similarity between the different timestamps. As shown in Figure 9, when the window size is set to 9, DNN-MVL can achieve the smallest filling error. As the window size increases, the filling accuracy decreases, but mainly remains stable. The reason is when considering longer temporal dependency, more parameters need to be learned. As a result, the training becomes harder and the filling error is bigger. Moreover, if considering single view, we cannot obtain the filling results. Due to considering the temporal, spatial and functional dependency, DNN-MVL has the best performance on filling the block missing values.

#### 6.4.3. Results of Filling Readings

**Figure 10:**
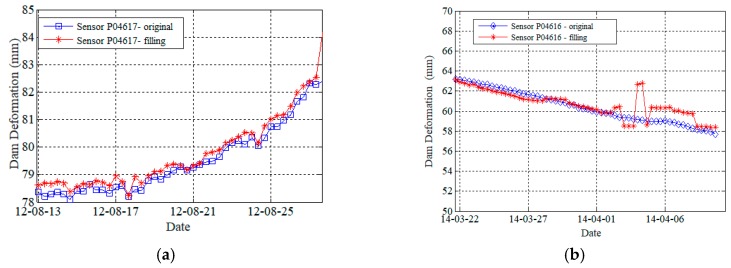
The illustration of filling results in different sensors’ readings. (**a**) Sensor P04617; (**b**) sensor P04616.

Figure 10 illustrates the results of filling the missing data in dam deformation data. Figure 10a,b are the filling results for the missing window size ω=45 and ω=60, respectively. The monitoring system generates the value six times in one day. The readings of sensor node P04617 from 12 August–28 August are missing, as shown in Figure 10a. Adopting the DNN-MVL method, the error of filling MAE is 2.38 and MSE was 3.25 when the window size ω was set to 45. Figure 10b plots the filling results of another sensor node P04616 with the window size ω=60 from 22 March–11 April. The MAE was 2.96 and MSE was 3.38. It can find that the bigger size of slide window ω may not always get the better filling results due to the missing readings of long sequence data.

#### 6.4.4. Results of Different Parameters

**Figure 11:**
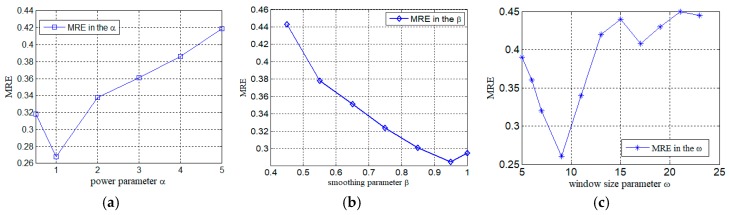
Impact of different parameters for DNN-MVL. (**a**) Power parameter α; (**b**) smoothing parameter β; (**c**) window size ω.

Different values of multiple parameters are tested to find a better setting for the final filling results. Figure 11a–c illustrates the filling results changing values of parameters from the different views, respectively. In the experiments, when the parameter α for the global spatial view is tuned to 1, the parameter β for the global temporal view is set to 0.95, the parameter ω of the window size for the local view was set to nine, respectively, the dam deformation has a minimum MRE. The optimal algorithm Adam [43] is adopted with a neural network of six fully connected layers. The number of hidden units are 64, 128, 256, 256, 64, and 32, respectively. The learning rate in a deep neural network is 0.001.

## 7. Conclusions

In this paper, a unified multi-view-learning model in a deep learning model (DNN-MVL) is proposed to fill the block missing values that jointly consider five views: global spatial view, global temporal view, local spatial view, local temporal view, and semantic view. These five views are modeled with inverse distance of weight interpolation, bidirectional simple exponential smoothing, user-based collaborative filtering, mass diffusion-based collaborative filtering with the bipartite graph, and structural embedding, respectively. The results of the five views are aggregated to a final value in a multi-view learning algorithm with DNN model to obtain the final filling readings. Experiments on large-scale real dam deformation data demonstrate that DNN-MVL has a mean absolute error about 6.5%, and mean relative error 21.4%, and mean square error 8.17% for dam deformation data, outperforming all of the baseline methods.

## Figures and Tables

**Table 1:** Statistics on missing values in experimental datasets.

Missing		Dam-Deformation
Block Missing	Spatial	2.7%
	Temporal (ω=11)	4.6%
General Missing		8.4%
Overall		15.2%

**Table 2:** Test dataset and ground truth partition.

Dataset	Dam Deformation
Start time	2012/01/01
End time	2017/08/14
Test set	March, June, September, December
Training set	others that are NOT in test set

**Table 3:** Comparison among different methods (based on dam deformation data).

Method	Spatial Block Missing			Temporal Block Missing		
	MAE	MRE	MSE	MAE	MRE	MSE
ARIMA	25.34	0.35	29.11	\	\	\
SARIMA	21.06	0.32	27.79	25.42	0.56	30.24
Kriging	\	\	\	15.31	0.28	27.85
DEMS	16.89	0.284	25.14	12.95	0.277	23.79
ST-KNN	17.55	0.31	28.87	12.32	0.27	22.59
CF	16.78	0.285	24.98	12.76	0.274	21.56
ST-MVL	14.62	0.256	21.62	11.51	0.23	19.47
DNN-MVL	13.54	0.19	19.45	9.75	0.156	18.38

**Table 4:** Results of different combination methods (dam deformation data).

Method		Spatial Block Missing			Temporal Block Missing		
		MAE	MSE	MRE	MAE	MSE	MRE
Global	IDW	\	\	\	12.64	21.12	0.242
	SES	16.31	23.68	0.32	\	\	\
	IDW + SES	16.31	23.68	0.32	12.64	21.12	0.242
Local	UCF	\	\	\	12.73	21.34	0.245
	MD-CF	15.98	22.14	0.294	\	\	\
	UCF + MD-CF	15.12	21.92	0.27	\	\	\
ST-MVL		14.62	21.62	0.256	11.51	19.47	0.21
DNN-MVL		13.54	19.45	0.19	9.75	18.38	0.156
